# A Computational Thermodynamics-Assisted Development of Sn-Bi-In-Ga Quaternary Alloys as Low-Temperature Pb-Free Solders

**DOI:** 10.3390/ma12040631

**Published:** 2019-02-20

**Authors:** Chih-han Yang, Shiqi Zhou, Shih-kang Lin, Hiroshi Nishikawa

**Affiliations:** 1 Department of Materials Science and Engineering, National Cheng Kung University, Tainan 70101, Taiwan; od6582@gmail.com; 2 Joining and Welding Research Institute, Osaka University, 11-1 Mihogaoka, Ibaraki, Osaka 567-0047, Japan; charleszhou1992@gmail.com (S.Z.); nisikawa@jwri.osaka-u.ac.jp (H.N.); 3 Hierachical Green-Energy Materials (Hi-GEM) Research Center, National Cheng Kung University, Tainan 70101, Taiwan; 4 Center for Micro/Nano Science and Technology, National Cheng Kung University, Tainan 70101, Taiwan

**Keywords:** Sn-Bi-In-Ga, low-temperature Pb-free solder, mechanical properties, CALPHAD, solidification

## Abstract

Low-temperature lead (Pb)-free solders are demanding in the electronic packaging industry, because it would open the door for various economic choices of polymeric materials as substrates and also revives the lower cost processes. Here, we proposed a tin–bismuth–indium–gallium (Sn-52.5Bi-2.68In-1Ga, SBIG (in wt.%)) quaternary low-temperature solder, designed based on systematic CALPHAD (CALculation of PHAse Diagram)-type thermodynamic calculations and corresponding key experiments. The solidification behavior of SBIG was carefully elaborated based on the computations using the lever rule and the Scheil model, and the experiments in terms of thermal analyses and microstructures of sample produced with step-quenching and various cooling rates. The mechanical properties of as-cast and 80 °C-annealed SBIG as well as their microstructures and fracture surfaces were evaluated in the tensile tests. The proposed SBIG solder is with a low liquidus temperature of 141.9 °C and is typically composed of the primary (Sn) phase, the (Sn) + (Bi) eutectic structure and a small amount of (Ga) phase. Air cooling has been identified as a satisfactory cooling rate, which would not lead to the formation of the brittle BiIn intermetallic compound. The as-cast SBIG solder exhibited high yield strength (YS) of 43.7 MPa, high ultimate tensile strength (UTS) of 53.3 MPa and an extremely large elongation of 97.3% as comparing to the conventional eutectic Sn-58Bi solder (YS: 43.1 MPa, UTS: 49.5 MPa, and elongation: 37.5%). However, the proposed SBIG solder does not possess qualified thermal stability, that significant degradation in both strength and elongation were observed after being subjected to extensive thermal ageing at 80 °C for 504 h.

## 1. Introduction

Soldering is one of the most important joining technologies for various levels of electronic packaging [1,2]. Tin (Sn)–based lead (Pb)–free solders have been introduced because of the health and environmental concerns upon the conventional Pb-Sn solders [3]. Several environmental-friendly Sn-based alloys such as Sn-3.0Ag-0.5Cu [4], Sn-14Bi-5In [5], Sn-0.7Cu [6], Sn-9Zn [7], Sn-8Zn-3Bi [8], and Sn-58Bi [9] have been considered the most promising candidates to replace the toxic Sn-Pb alloy from electronic packaging systems [10]. In order to avoid thermal damages in step-soldering processes and to eliminate the coefficient of thermal expansion (CTE) mismatches of materials in packaging modules, low-temperature Pb-free solders with low cost and high reliability are demanding in the electronic industry [7,11,12]. The Sn-58 wt.% Bi (Sn-58Bi) eutectic alloy with high mechanical properties, good wettability and low melting temperature at 139 °C has drawn great attentions in the industry [13,14]. However, the brittle nature of the Bi-rich phase and microstructure coarsening during thermal aging is a significant issue in employing the Sn-58Bi solder [11,15,16]. Therefore, designing proper alloying elements for improving the mechanical properties of the Sn-58Bi solder is necessary for low-temperature soldering applications. In other words, the goal of alloy design is to improve the elongation of Sn-Bi-based solders by doping, while keeping their low melting temperatures.

Minor doping to adjust the properties of Sn-Bi alloys has been extensively studied [12,13,14,15,16,17,18,19,20,21]. For instance, it was found that 0.1 wt.% Cu addition in Sn-40Bi solders resulted in an increase in both ultimate tensile strength (UTS) and ductility, while 2 wt.% Zn addition in Sn-40Bi-0.1Cu could further enhance UTS, but decreased the elongation remarkably [17]; 0–2 wt.% Zn addition could increase the UTS and effectively suppressed the coarsening of (Bi) phase in Sn-Bi solders [18,19]; 0.5 and 1 wt.% nickel (Ni) addition improved the ductility of eutectic Sn–58Bi [15]; 0.25–0.5 wt.% Ag addition in near-eutectic Bi–45Sn alloys improved their ductility [20]; and among Ag, Cu, Zn, and antimony (Sb) as the doping elements for Sn-Bi alloys, 0.5 wt.% Ag addition increased the tensile strength the most, while 0.5 wt.% Cu and Sb improved the elongation significantly but changed the tensile strength slightly [21]. In particular, Chen et al. [13] showed that a proper amount of In addition in the eutectic, the Sn-58Bi alloy could not only lower the melting temperature, but also improve the elongation. However, with an excess amount of In addition, the brittle BiIn intermetallic compound (IMC) appeared in the microstructure, which dramatically deteriorated the mechanical properties. Based on a trial-and-error approach, 2.5 wt.% In addition was proposed to be an optimal doping level. At last, Lin et al. [12] reported that a minor amount of gallium (Ga) addition in Sn-58Bi solders would lead to the formation of the γ-Cu_9_Ga_4_ IMC at the interface with Cu substrates, acting as a ‘native diffusion barrier’ to effectively suppress the interfacial IMC growth. 

It is evident that the microstructure and mechanical properties of Sn-Bi-based solder could be significantly manipulated with minor alloying elements. The positive alloying effects can generally be categorized into two kinds as follows. Firstly, the alloying effects may induce solidification of phases with higher liquidus temperature, such as (Zn), Ag_3_Sn, Cu_6_Sn_5_, Ni_3_Sn_4_, and SnSb, which act as extra heterogeneous nucleation sites and prohibit grain growth of both (Sn) and (Bi) phases [15,17,18,19,20,21]. The resultant refined microstructure could neutralize the brittle property of (Bi) phase and consequently exhibit better mechanical properties. The other type of alloying elements is with lower melting temperatures, such as In, which usually lowers the melting point, and softens the (Sn) phase and remarkably enhances the elongation. However, the doping level of either type of alloying elements has to be carefully designed and controlled to avoid the undesired alloying effects, namely the formation, overgrowth, and aggregation of brittle IMCs. The grain sizes of the major (Sn) and (Bi) phases, and the amount and distribution of the minor other phases in the solder matrix have to be carefully optimized for a compromised mechanical property. In this study, alloy design based on computational thermodynamics-assisted experiments is demonstrated to optimize the chemical composition of low-temperature Pb-free solder, and a Sn-Bi-In-Ga quaternary alloy is proposed.

## 2. Materials and Methods 

### 2.1. CALPHAD Thermodynamic Modeling

Thermodynamic calculations based on the CALPHAD (calculation of phase diagram) method were performed using the PANDAT software [22] with the Sn–Bi–In ternary thermodynamic model [23]. Phase equilibria of the Sn–Bi–In ternary system, including isothermal sections, liquidus projections, and solidification paths, were calculated for elucidating the resultant phase transformation and microstructures.

### 2.2. Solder-Alloy Preparation and Ageing Process

The designed Sn–52.5Bi–2.68In–1Ga alloy (in wt.%) was prepared with a commercially available Sn–58Bi alloy (99.95%, Alfa Aesar, Tewksbury, MA, USA) and pure Sn (99.99%, Alfa Aesar, Tewksbury, MA, USA), In (99.9%, Alfa Aesar, Tewksbury, MA, USA) and Ga (99.9%, Alfa Aesar, Tewksbury, MA, USA). Proper amounts of the constituent materials were weighted and homogenized at 400 °C in the alumina crucible for 50 h under the protection of high-purity argon atmosphere and then cooled down in the high-purity argon atmosphere. After homogenization, the alloys were subsequently remelted at 250 °C for 1 h before casting. The alloys were cast with water quenching, air cooling, and furnace cooling, respectively, for investigating the effect of cooling rates. Some of these as-cast solder bulks were subsequently immersed in an oil bath at 80 °C for 504 h for thermal aging studies.

### 2.3. Microstructural Characterizations, Thermal Analyses, and Tensile Tests

**Figure 1:**
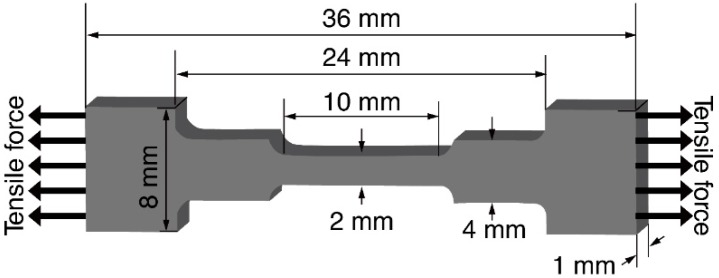
Schematic illustration of the tensile test specimen.

The polished cross-sectional microstructures of the as-cased and aged alloys as well as the morphology of fracture surfaces were observed using a field-emission scanning electron microscope (FE-SEM, SU-70, Hitachi, Japan). The chemical composition determinations and elemental mappings were performed using a field-emission electron probe micro-analyzer (FE-EPMA, JXA-8530F, JEOL, Japan). Differential scanning calorimetry (DSC, STA-449-F3, Netzsch, Germany) was employed to determine the thermal behavior of the alloys. Prior to thermal analyses, the alloys were annealed at 100 °C for 72 h to eliminate the effect of non-equilibrium solidification at a finite cooling rate during casting. Samples of approximately 10 mg were prepared, sealed in an aluminum pan, and examined under nitrogen atmosphere. These samples were heated to 170 °C at a heating rate of 1, 2, 3, 5 and 10 °C/min, respectively, kept at 170 °C for 5 min to ensure the thermal equilibrium of molten alloys before the cooling processes, and then, the samples were cooled down to the room temperature at the same rates as in their heating processes. For the tensile tests, the alloys were re-melted at 250 °C for 2 h in a crucible and cast into bar-shaped steel molds. After that, the bar-shaped ingot was machined into dumbbell shape specimens according to the American Society for Testing and Materials (ASTM) standard for tensile tests as shown in Figure 1. Tensile tests were performed at room temperature on a Shimadzu Autograph AG-X machine at a strain rate of 0.3 mm/min. to obtain data on the tensile properties, i.e., the ultimate tensile strength (UTS), yield strength (YS), elongation, and Young’s modulus. For each tensile testing condition, three measurements were performed and the average value and the corresponding uncertainty were obtained.

## 3. Results and Discussion

### 3.1. Alloy Design

**Figure 2:**
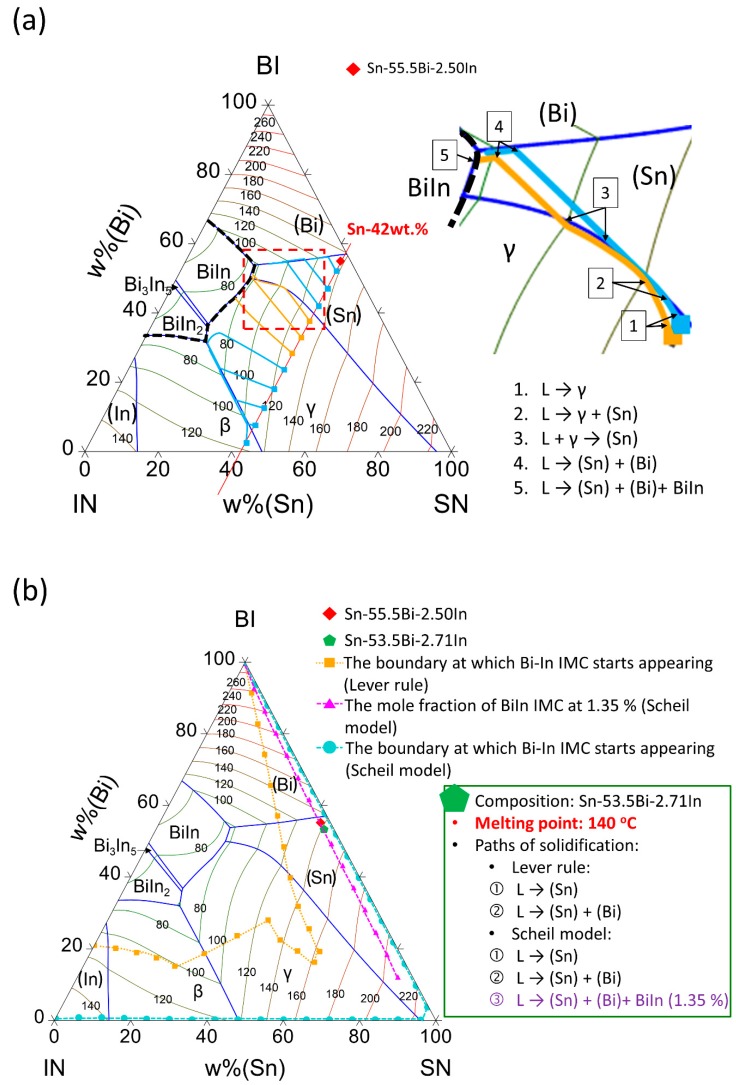
(**a**) The calculated liquidus projection of the Sn-Bi-In ternary system with liquidus isotherms every 20 °C. (**b**) The boundary at which BiIn intermetallic compound (IMC) starts appearing based on the lever rule and the Scheil model, and the 1.35 % mole fraction of BiIn IMC based on the Scheil model.

Figure 2a shows the calculated liquidus projection of the Sn-Bi-In ternary system with liquidus isotherms every 20 °C. The thin deep blue lines are the univariant lines, and the intersections of univariant lines are the invariant points. The lowest liquidus temperatures involve the Bi–In IMCs below 80 °C. Firstly, we performed a virtual experiment by considering the alloy compositions examined in the literature [16], in which the content of Sn was fixed at 42 wt.%, while the content of In was increased from the based Sn-58Bi with 5 wt.% as interval. The eleven compositions, namely Sn-53Bi-5In, Sn-48Bi-10In, Sn-43Bi-15In, Sn-38Bi-20In, Sn-33Bi-25In, Sn-28Bi-30In, Sn-23Bi-35In, Sn-18Bi-40In, Sn-13Bi-45In, Sn-8Bi-40In, and Sn-3Bi-45In, are shown as squares in Figure 2a. The solidification of each alloy composition was calculated based on the Lever rule. The solidification paths of the alloys, i.e., evolutions of the liquid phase composition during descending temperatures, are superimposed on Figure 2a. The solidification paths indicate each step of precipitation as well as the final existing phases. Two types of solidification paths near the Sn-Bi binary boundary in the red dashed region are highlighted in close-up with orange and light blue lines. For the blue one, the γ phase precipitates primarily, followed by the co-precipitation of the γ and (Sn) phases. Then, after a peritectic-type reaction, only the (Sn) phase is formed. Finally, the solidification terminates with the (Sn) + (Bi) eutectic reaction along the univariant line, resulting in (Bi) and (Sn) being the final existing phases. For the orange one, there are five solidification steps. The first four steps are identical as the blue one. However, the fifth step involves a Class I reaction: L = (Sn) + (Bi) +BiIn, so the undesired brittle BiIn IMC formed. Based on the solidification analyses of all the alloys, the phase compositions, particularly the existence of Bi-In IMCs, can be revealed based on the computational thermodynamic approach.

The two goals for improving the conventional binary Sn-Bi eutectic solders are clearly defined in the alloy design at the first place, namely (1) enhancing the elongation property, while (2) keeping the melting (liquidus) temperature below 140 °C. In order to optimize the alloying mount of In content in the Sn-Bi-In solder, the solidification behavior of entire Sn-Bi-In ternary compositional space was evaluated with a similar process, i.e., checking the solidification paths of alloys with various Bi/In ratios with fixed Sn contents every 6 wt.% Sn as an interval. As shown in Figure 2b, the compositional threshold of formation of Bi-In IMCs during solidification was evaluated based on the lever rule and the Scheil model, and the boundaries of Bi-In IMC formation during solidification based on both solidification models were superimposed on the Sn-Bi-In liquidus projection as the orange dotted line and the light blue dashed line, respectively. For the equilibrium condition based on the lever rule (orange dotted line), a quite broad compositional area could meet the criterion of no Bi-In IMC precipitation; however, for the non-equilibrium condition based on Scheil model (light blue dashed line), only a very narrow window near the Sn-In and Sn-Bi binary boundaries could meet this criterion. Since the alloy solidification in real soldering processes should lie between these two extreme cases, the optimal alloying level should also lie between the two boundaries when considering the finite rate of diffusion. As reviewed in the Introduction section, a trial-and-error experiment suggested that the Sn-55.5Bi-2.5In solder could fulfill elongation requirement without the formation of the brittle Bi-In IMCs [16]. Therefore, this proposed composition was taken to perform solidification calculation based on the Scheil model, and the resultant final solidified phases consisted of 1.35% BiIn. This suggests that 1.35% BiIn phase in Scheil solidification can be considered as the optimal alloying level with the most intake of In in the solidified solution phases without Bi-In IMC formation in reality. With the 1.35% BiIn in Scheil solidification being the criterion to meet the first goal on elongation, as shown in Figure 2b, the pink dashed line superimposed on the Sn-Bi-In liquidus projection illustrates the desired In-alloying levels. Then, with the consideration of the other goal of a 140 °C melting temperature, the nominal composition of the desired solder was proposed to be Sn-53.5Bi-2.71In as shown in Figure 2b. 

Interfacial properties are crucial for the reliability of solder joints [1,12,24,25]. Particularly, for Sn-Bi-based solders, Sn preferentially reacts with the commonly used substrates in electronic packaging, e.g., Cu, while Bi is usually unreactive as an inert, so after reactions, Bi is excess and left behind at the interfaces, which would deteriorate the joint strength and ductility. It has been reported in our previous work [12] that a minor amount of Ga doping in Sn-Bi solders could effectively suppress the interfacial reactions. This is because Ga segregates towards the interfaces during soldering and predominantly reacts with Cu substrates to form the Cu_9_Ga_4_ phase with negligible Sn solubility, which could act as a native diffusion barrier between Sn and Cu. Therefore, 1 wt.% Ga was added to the proposed ternary Sn-53.5Bi-2.71In solder. Finally, a quaternary Sn-52.5Bi-2.68In-1Ga (SBIG) solder is proposed, in which In and Ga were added to improve the mechanical property of solder bulks and joint interfaces, respectively.

### 3.2. DSC Measurements

**Figure 3:**
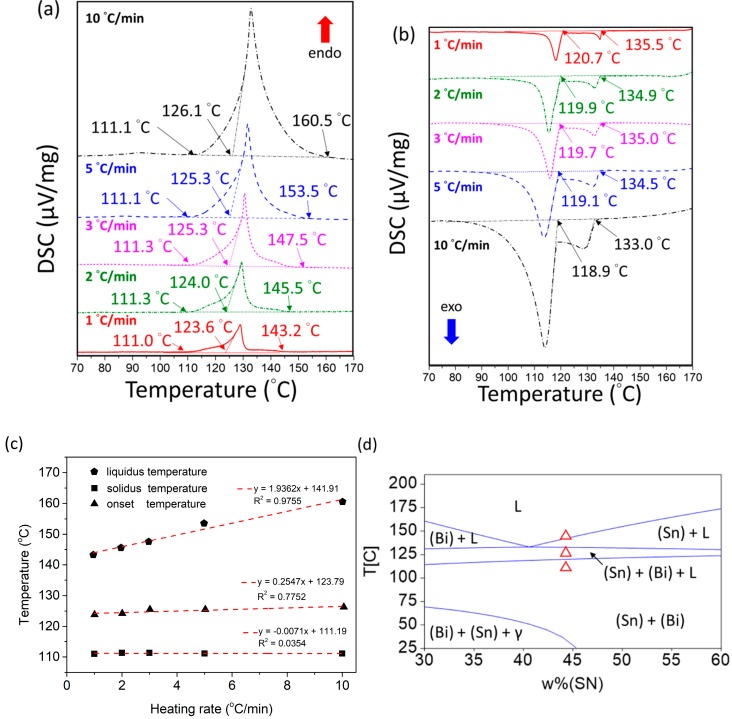
The differential scanning calorimetry (DSC) curves of 1, 2, 3, 5, and 10 °C/min in (**a**) the heating process and (**b**) the cooling process of the Sn-52.5Bi-2.68In-1Ga (SBIG) alloys. (**c**) The linear extrapolations of *T_S_*, *T_0_*, and *T_L_*. (**d**) The calculated isoplethal section of Sn–53.5Bi–2.71In with superimposed *T_S_*, *T_0_*, and *T_L_*.

The melting temperature and the solidification behavior are critical characteristics for low-temperature solders. As shown in Figure 3a,b, the proposed SBIG quaternary alloy was subjected to thermal analyses using a DSC with various heating and cooling rates at 1, 2, 3, 5 and 10 °C/min, respectively. Prior to each heating process, the sample was equilibrated at 100 °C for 72 h to ensure the initial equilibrium condition, and for a similar reason, the sample was held at 170 °C for 5 min prior to each cooling process. 

In the heating processes as shown in Figure 3a, the solidus temperature (*T_S_*), an onset temperature (*T_0_*), and liquidus temperature (*T_L_*) are observed. A weak but finite tail-off after each endothermic peak is shown in Figure 3a, which is higher than the onset temperatures. As pointed out by Ghosh et al. [26], Speyer [27], and Yoon et al. [28], the measured liquidus temperature (*T_L_*) would be the same as the onset temperature (*T_0_*) without the tailing behavior, such as in a eutectic alloy. For such cases, all the solid phases would transform to the liquid phase completely and simultaneously at the eutectic temperature and composition, so the measured *T_S_* would depend linearly on the heating rates and *T_0_* would be identical to *T_L_* [28]. However, for alloys with tailing such as in the off-eutectic SBIG alloy, the two phase (Sn) + (Bi) cannot transform to liquid simultaneously and the *T_L_* is not *T_0_*. Due to the multiple reactions during heating in the off-eutectic SBIG alloy, the heating curves are with multi-slopes involving corresponding uni-variant and invariant reactions as compared with the single slope in invariant eutectic point. This multi-slopes phenomenon is more pronounced at higher heating rates. Five heating and cooling rates were carried out, and the obtained characteristic temperatures at each heating rate were linearly extrapolated to minimize the effect of finite rate of heat transfer, as shown in Figure 3c. The *T_S_*, *T_0_* and *T_L_* are determined to be 111.2, 123.8, and 141.9 °C, respectively, which are superimposed on the calculated Sn–53.5Bi–2.71In isoplethal section as shown in Figure 3d. Despite the calculated isopleth without considering the minor Ga doping, the calculated reactions temperatures generally agree with the experiments and present the three reactions in the order of descending temperature:

(Sn) + L → L           at 141.9 °C(1)

(Sn) + (Bi) + L → (Sn) + L      at 123.8 °C(2)

(Sn) + (Bi) → (Sn) + (Bi) + L     at 111.2 °C(3)

In the cooling process as shown in Figure 3c, two exothermic peaks were observed and they represent the solidification processes:

L → (Sn)(4)

L + (Sn) → (Sn) + (Bi)(5)

As shown in the cooling curves, the onset temperatures of the first solidification reaction, namely liquid → (Sn) with 1, 2, 3, 5 and 10 °C/min-cooling rates were 135.5, 134.9, 135.0, 134.5, and 133.0 °C, respectively. On the other hand, the onset temperatures of the second solidification reaction, namely liquid + (Sn) → (Sn) + (Bi) with 1, 2, 3, 5 and 10 °C/min-cooling rates were 120.7, 119.9, 119.7, 119.1, and 118.9 °C, respectively. Based on the equilibrium reaction temperatures obtained from heating experiments, the equilibrium solidification temperatures for the reactions (4) and (5) were 141.9 and 123.8 °C, respectively. Comparing the onset temperatures of cooling curves with the equilibrium reaction temperatures, the higher cooling rates result in larger extent of undercooling. The extents of undercooling for the first and the second solidification reactions were 6.4–8.9 and 3.1–4.9 °C, respectively. As expected, the undercooling in the second solidification was less significant than that of the first one, because the solid (Sn) phase formed in the first reaction could act as the heterogeneous nucleation sites for the second reaction. 

### 3.3. Microstructure

#### 3.3.1. As-Cast and Step-Quenched Samples

**Figure 4:**
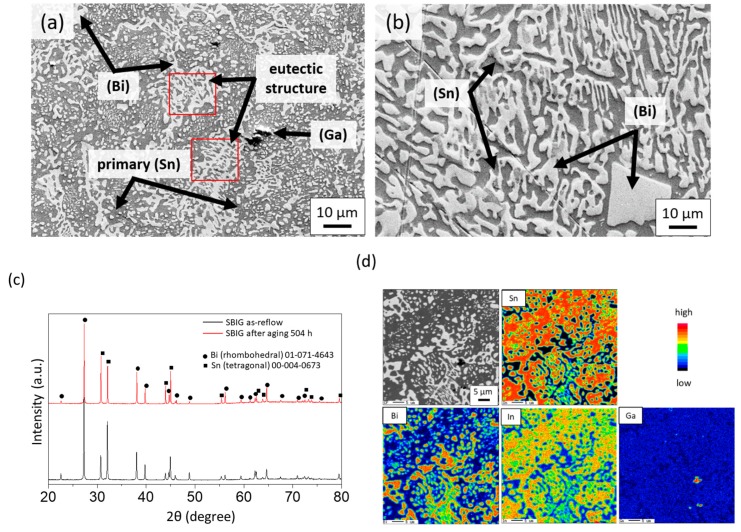
The field-emission scanning electing microscope (FE-SEM) images of (**a**) as-cast SBIG and (**b**) as-cast Sn–58Bi alloys. (**c**) The XRD patterns of as-cast and 80 °C and 504 h-aged SBIG alloys. (**d**) The field-emission electron probe micro-analyzer (FE-EMPA) elemental mappings of the as-cast SBIG alloy.

Figure 4a,b shows the microstructures of as-cast SBIG and Sn-58Bi alloys prepared by air cooling, respectively. For the as-cast SBIG alloy, the bright, grey, and dark regions under the backscattered electron (BSE) detector are the Bi-, Sn-, and Ga-rich phases, respectively. The composition of Ga-rich phases was determined to be Sn–4.11 wt.% Bi–0 wt.% In–92.59 wt.% Ga using energy dispersive spectroscopy (EDS). A similar phase formation was found in the as-cast Sn-58Bi alloy except for the dark Ga-rich phase. Comparing the microstructures, much finer grains and more Sn-rich phase were found in the SBIG solder than in the Sn–58Bi solder. Figure 4c shows the X-ray diffraction (XRD) pattern of the as-cast SBIG alloy. The major phases are the (Bi) and (Sn) phases, which is in accordance of the observation in BSE microstructure. Since there is no IMC in the Ga–Sn, Ga–Bi, and Ga–In binary systems, the minor Ga-rich phase observed in SBIG was likely an unreacted (Ga) phase, which is a liquid phase at room temperature and is invisible in the XRD analysis. The three phases presented in the SBIG alloy are all elementary phases, namely the (Sn), (Bi), and (Ga) phases. The elemental-mapping analysis of SBIG is shown in Figure 4d. It was found that Sn essentially does not dissolve in the (Bi) phases; however, In and Ga are partially dissolved in the (Bi) phase. In addition, there are some amounts of In, Ga, and Bi dissolved in the (Sn) phase. Bi-rich phase contains some part of In and Ga. The (Ga) phase distributes in between the major (Sn) and (Bi) phase regions in terms of micron to submicron regions. The proposed SBIG exhibits similar phases as the eutectic Sn-Bi alloy except for well-distributed (Ga) micro-regions and much finer grains in the solder matrix.

**Figure 5:**
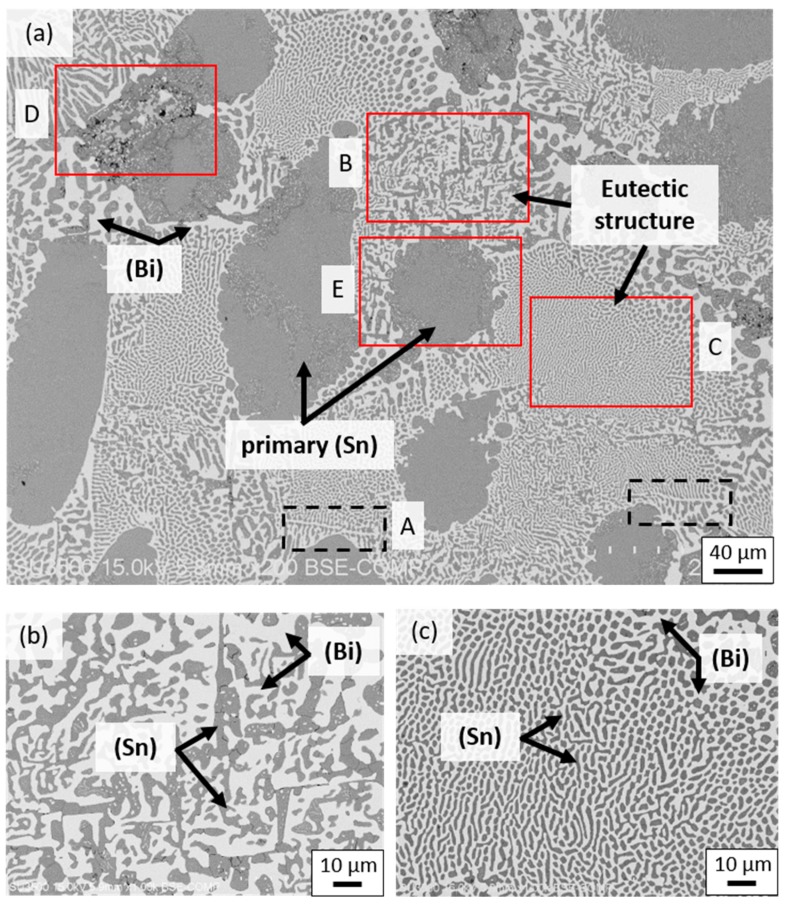

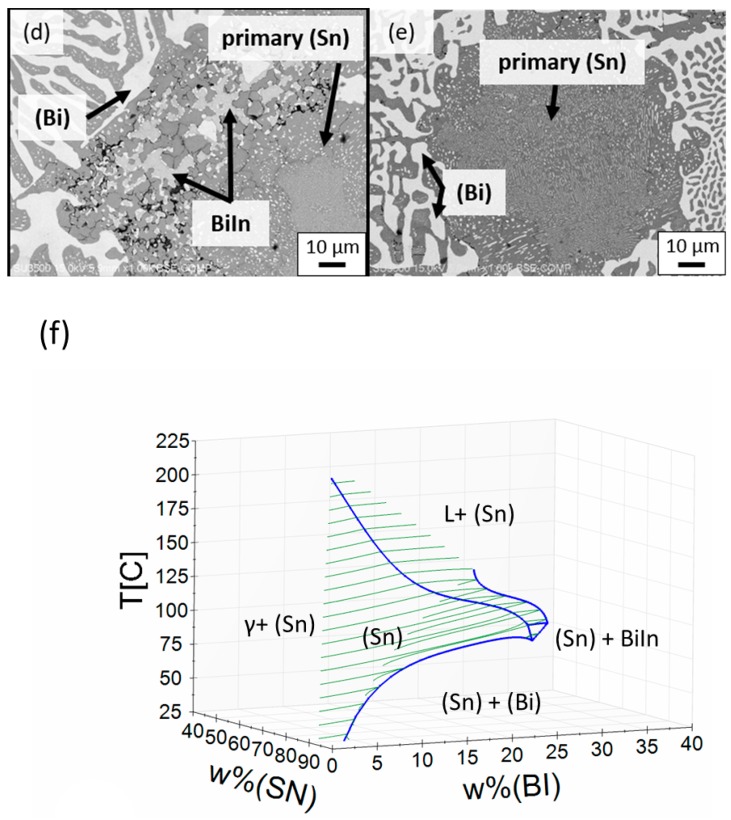
(**a**) The SEM images of the “step-quenched” SBIG alloy. The close-ups at the (**b**) coarse eutectic, (**c**) fine eutectic, (**d**) BiIn IMC, and (**e**) primary (Sn) phase regions. (**f**) The three-dimensional (3D) space diagram of the single (Sn) phase region in the Sn-Bi-In ternary system.

While the phase formation was rather simple in SBIG, its striking different microstructure as compared with Sn-58Bi solder was formed through the solidification reactions at similar temperatures, which induce the complexity in determination of the primary solidification phase and the following microstructure formation. The “step quenching” experiments were performed using a DSC to investigate the mechanism of microstructure formation. Figure 5a shows the microstructure of “step quenched” SBIG, which was cast and naturally cooled to 126 °C, and subsequently annealed at this temperature for 3 h before fully solidified. The holding temperature at 126 °C in the “step quenching” experiment allowed the primarily solidified phase to grow and to equilibrate with the neighboring liquid phase prior to the precipitation of other phases. As shown in Figure 5a, the grey (Sn) phase is significantly larger than the other phase, which confirms that the (Sn) phase is the primary solidification phase in SBIG. The other regions are likely the liquid phase prior to the sample that was quenched from 126 °C. As indicated in the liquid projection and solidification path shown in Figure 2b, the following reaction involves co-precipitation of (Sn) and (Bi) phases. It can be seen that fishbone-like (Bi) dendrites appeared adjacent to the primary (Sn) phase regions as highlighted with black dashed box shown in Figure 5a. This was likely caused by the accumulation of Bi in the liquid phase after the precipitation of primary (Sn) phase. This trend can also be seen from the slope of the solidification path shown in Figure 2a, in that the Bi content in the liquid phase goes up during the primary (Sn) phase precipitation. Hence, the regions enriched in Bi surrounding the primary (Sn) phase were presumed to be the immediate following solidified regions. The following uni-variant (Sn)+(Bi) co-precipitation exhibited coarse and fine lamellar eutectic structures, as the close-ups shown in Figure 5b,c, respectively. The two kinds of lamellae were formed associated with thermal instability due to local heat flows in non-equilibrium solidification [29]. The fine eutectic structure was formed after the neighboring coarse eutectic structure. Nevertheless, the average composition in the two eutectic structures was nearly identical and was determined to be Sn–56 wt.% Bi–6 wt.% In–3 wt.% Ga. As the (Sn) + (Bi) co-precipitation proceeded, the liquid phase was significantly more enriched in In, as shown in Figure 2. Consequently, the BiIn IMC may precipitate from the In- and Bi-rich liquid phase as shown in the close-up shown in Figure 5d. Its composition was determined to be Sn–44.15 wt.% Bi–47.87 wt.% In–3.16 wt.% Ga using EDS. While the SBIG alloy mostly solidified at the (Sn)+(Bi) co-precipitation, solid-state transformation proceeded during the cooling process. As shown in Figure 5e, nano ellipsoid (Bi) grains were found within the primary (Sn) phase. Figure 5f shows the three-dimensional (3D) space diagram of the single (Sn) phase region in the Sn-Bi-In ternary system. The formation of nano (Bi) grains can be attributed to the decreasing solubility of Bi in the (Sn) phase in descending temperature, so the supersaturated Bi in the (Sn) phase was homogeneously ejected out which uniformly nucleated as nano (Bi) grains.

#### 3.3.2. Effects of Cooling Rates and Ageing on Microstructure

**Figure 6:**
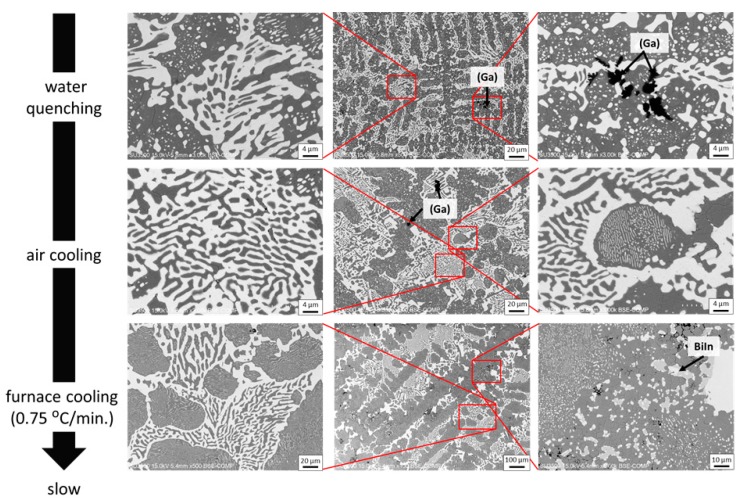
The microstructure of the SBIG solder cast with various cooling rates ranging from the fastest water quenching, air cooling, to the slowest furnace cooling.

Figure 6 shows the microstructure of the SBIG solder cast with various cooling rates ranging from the fastest water quenching, air cooling, to the slowest furnace cooling (ca. 0.75 °C/min). The solidification behavior and phase formation of these samples generally follow the detailed discussion in the previous sessions. However, besides the expected large difference in grain size in the samples cast with different cooling rates, the distribution of (Ga) phase and the formation of BiIn strongly depend on the cooling rate. For the distribution of (Ga) phase, it is found that the faster the cooling rates are, the higher the chance to find the (Ga) phase in the matrix. Since the (Ga) phase remained as the liquid phase till the end of the solidification of the major solid phases, i.e., the (Sn) and (Bi) phases, the presence of (Ga) phase in the matrix is actually encapsulated in the matrix by the solidified (Sn) and (Bi) phases. When the cooling rate is fast enough, the (Ga) phase can be kept in the matrix before being ejected to the bulk surface. However, for the cases of slow cooling rates, it is likely the remaining liquid phase could always consolidate driven by surface tension. Therefore, the (Ga) phase could rarely be seen in the slowly cooled samples. As for the formation of the BiIn IMC, it was also observed in the slower cooled samples, i.e., furnace cooling one. As discussed in the previous sessions, the formation of BiIn IMC is thermodynamic favorable in equilibrium solidification (lever rule) while it may be kinetically prohibited with a finite cooling rate. As a result, in the samples with slower cooling rates, the corresponding solidification may proceed further until the formation of BiIn IMC. In summary, a sufficient fast cooling rate is necessary to produce the desired microstructure as designed. Nevertheless, the most typical air cooling process already satisfied the optimal cooling rate. 

**Figure 7:**
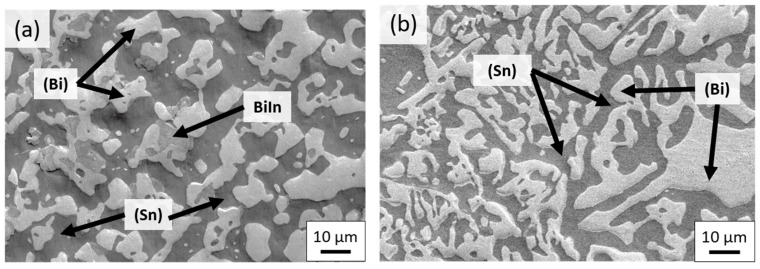

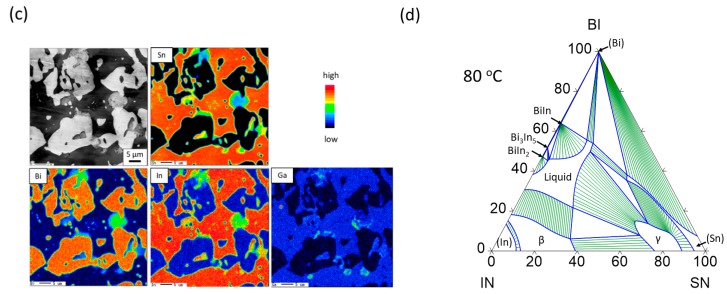
The microstructures of (**a**) SBIG and (**b**) Sn-58Bi solders, respectively, that were cast with air cooling and thermally aged at 80 °C for 504 h. (**c**) The EMPA elemental mappings of the SBIG after being aged at 80 °C for 504 h. (**d**) The 80 °C isothermal section of the Sn-Bi-In ternary system.

Figure 7a,b show the microstructure of SBIG and Sn-58Bi solders, respectively, that were cast with air cooling and thermally aged at 80 °C for 504 h. As compared with the as-cast samples shown in Figure 4a,b, the eutectic structure in both solders coarsened, which is known to deteriorate the mechanical properties [15]. For the SBIG solder, not only was the eutectic structure significantly coarsened, but also a new light gray phase formed as shown in Figure 7a. Figure 7c shows the elemental-mapping of the SBIG sample based on EPMA. The new light gray phase is composed of all the constituent elements, Bi, In, Sn, and Ga. Besides, its composition is not homogeneous after the prolonged ageing, indicating it is not an equilibrated phase at 80 °C. Instead, it was likely a liquid phase at the ageing temperature and solidified at the end of thermal ageing. As shown in the 80 °C isothermal section of the Sn-Bi-In ternary system (Figure 7d), the ternary Sn-Bi-In liquid with the composition of Sn-54.0 wt.%Bi-25.6 wt.% In is in equilibrium with both (Bi) and (Sn) phases. Since the addition of Ga would further reduce the liquidus temperature [30], it is evident that the quaternary Sn-Bi-In-Ga liquid phase presented at 80 °C and solidified into BiIn IMC after ageing. As shown in the Ga- and Sn-mapping in Figure 7c, it is likely the BiIn IMC nucleated on the (Bi) phase and the final liquid phase enriched in Ga and Sn finally solidified near the (Sn) phase. In summary, neither the SBIG nor the Sn-58Bi solders possessed qualified thermal stability. Brittle BiIn IMC and even semi-solid state may form in SBIG during thermal ageing. The semi-solid structure would act like voids in the alloy, thus, it can be speculated that this phase transformation will deteriorate the mechanical properties significantly. Further alloy design is needed to preserve the superior microstructure in as-cast SBIG under thermal ageing.

### 3.4. Mechanical Properties and Deformation Behavior

**Figure 8:**
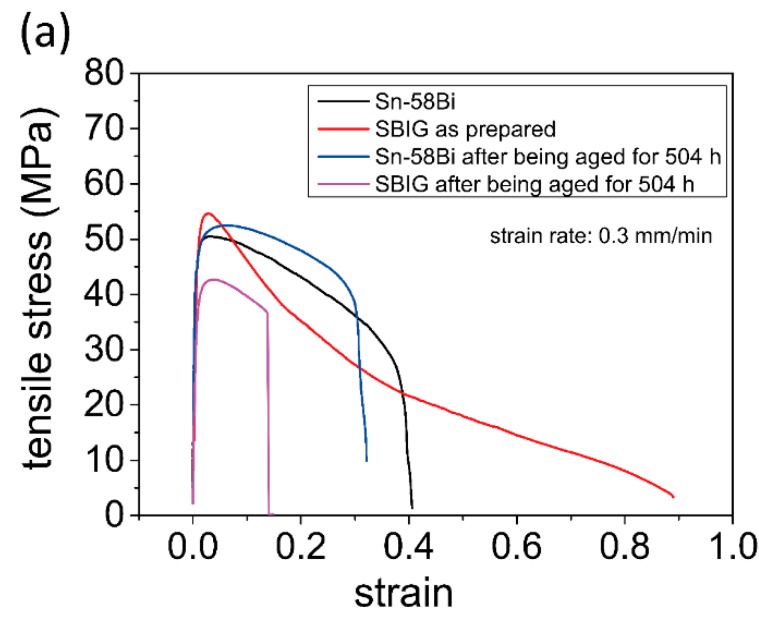

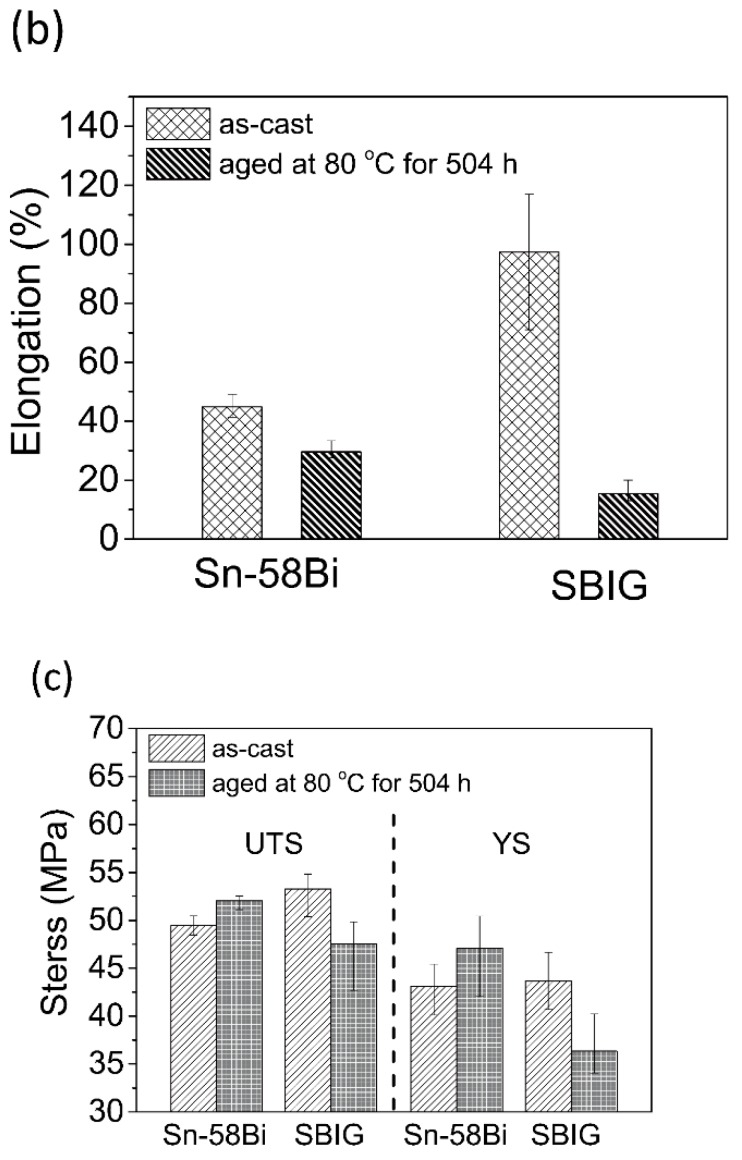
(**a**) The stress-strain (SS) curves of the as-cast and 80 °C-annealed eutectic Sn–58Bi and SBIG samples in tensile tests, respectively. (**b**) The elongation, and (**c**) ultimate tensile strength (UTS) and yield strength (YS) of as-cast and 80 °C-aged eutectic Sn58Bi and SBIG samples.

Figure 8a shows the stress-strain (SS) curves of the as-cast and 80 °C-annealed eutectic Sn–58Bi and SBIG samples in tensile tests, respectively. The SS curve of the as-cast SBIG was not uniform after the load reached UTS; this can be attributed to the minor Ga-rich liquid phase retained in the as-cast SBIG alloy. Nevertheless, the elongation, ultimate tensile strength (UTS), and yield strength (YS) of the four samples can be obtained from the SS curves and summarized in Figure 8b,c, respectively. As shown in Figure 8b, the elongation of as-cast SBIG and Sn–58Bi are 97.3% and 37.5%, respectively. The elongation of SBIG is more than doubled larger as compared with that of Sn–58Bi solders. However, the elongation of both samples decreased after thermal ageing, especially for SBIG. As shown in Figure 8c, the UTS and YS of as-cast SBIG samples are slightly higher those of as-cast Sn-58Bi samples; however, the UTS and YS of 80 °C-annealed SBIG samples become much lower those of Sn-58Bi samples after aging. 

**Figure 9:**
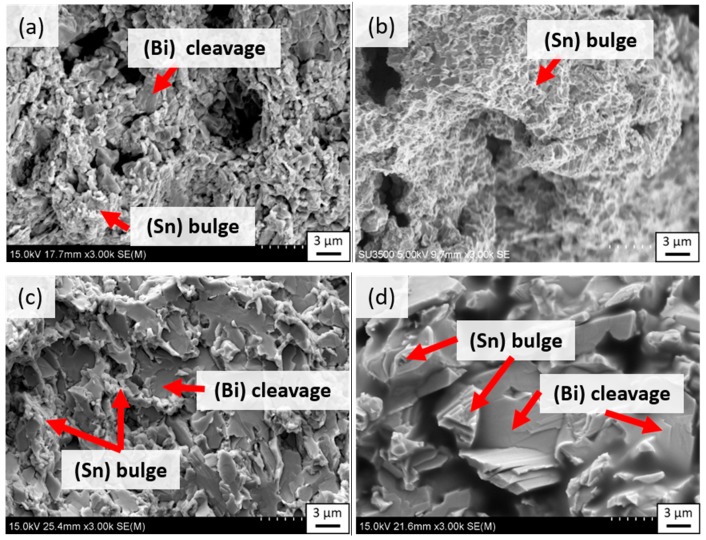
Fracture surfaces of (**a**) as-cast SBIG, (**b**) as-cast Sn–58Bi, (**c**) 80 °C-annealed SBIG, and (**d**) 80 °C-annealed Sn–58Bi samples.

Figure 9a–d shows fracture surfaces of as-cast and 80 °C-annealed eutectic Sn–58Bi and SBIG samples after tensile testing, respectively. As shown in Figure 9a, the fracture surface of Sn–58Bi samples before ageing exhibited a mixed pattern with bulge and cleavage appearances, indicating a low ductility. It has been reported that the cleavage morphology was from the fractured brittle (Bi) phase, and the bulge structure was from the ductile (Sn) phase [18]. As shown in Figure 9b, the bulge structure dominated the fracture surface of SBIG samples, which is consistent with its great ductility shown in Figure 9a,b. After thermal ageing at 80 °C for 504 h, as shown in Figure 9c, the number of cleavage fractured phases greatly increased and that of bulge phases correspondingly decreased in the Sn–58Bi samples. For the aged SBIG, as shown in Figure 9d, a coarsened microstructure with the predominant cleavage structure reflects the fact of its low UTS, YS, and elongation after ageing as shown in Figure 8.

As compared with the conventional as-cast eutectic Sn–58Bi solders, the proposed as-cast SBIG solder exhibits much finer the microstructure, which is known to be inherently related to stronger UTS and better elongation in tensile tests [15,31]. In addition to the refined microstructure, the superior elongation properties in SBIG also resulted from the fact that there was a larger proportion of the (Sn) phase in microstructure and a significant amount of dissolved In in the (Sn) phase. This compensated the strengthening effect of dissolved Bi, and thus softened the (Sn) phase [16], as shown in the microstructures and EPMA elemental mappings. As a synergetic consequence, as shown in Figure 8a,b, the elongation of the as-cast SBIG was remarkably improved. However, for the solders that were thermally aged, again as comparing to the conventional eutectic Sn-58Bi solders, the SBIG solder exhibited more significant degradation in mechanical properties, which had to do with the grain coarsening and the formation of the brittle BiIn IMC as previously discussed. In conclusion, the proposed SBIG solder has a satisfactory liquidus temperature for the soldering process with thermally sensitive polymer-based substrates, and its superior microstructure and mechanical properties in the as-cast condition make it a good candidate for low-temperature applications, e.g., biomedical and wearable devices. However, it is not suitable to be used in power- or heat-demanding environments where mechanical properties may significantly degrade.

## 4. Conclusions

A Sn–52.5Bi–2.68In–1Ga (SBIG) quaternary alloy was proposed based on the criteria of low liquidus temperature around 140 °C and no formation of brittle Bi-In IMCs using CALPHAD-type thermodynamic modeling based on the lever rule and the Scheil model. Several conclusions can be drawn as follows:

- The microstructure of the proposed SBIG solders was significantly different from the conventional eutectic Sn-58Bi solder, which is composed of the primary (Sn) phase, the (Sn)+(Bi) eutectic structures, and minor sub-micron to micron (Ga) phase.
- If the SBIG solders were subjected to a slower casting process, the undesired brittle BiIn IMC would form and the (Ga) phase may aggregate together instead of dispersing in the solder matrix. Nevertheless, the rate of air cooling has satisfied the optimal cooling rate for desired microstructure.
- The *T_S_*, *T_0_*, and *T_L_* of SBIG were determined to be 111.2, 123.8, and 141.9 °C, respectively, and the undercooling of SBIG ranges from 6.4 to 8.9 °C under slow furnace cooling to fast water quenching.
- The as-cast SBIG exhibits high YS of 43.7 MPa, high UTS of 53.3 MPa, and an extremely large elongation of 97.3% as comparing to the conventional eutectic Sn-58Bi solder (YS: 43.1 MPa, UTS: 49.5 MPa, and elongation: 37.5 %). However, the proposed SBIG solder did not possess qualified thermal stability, in that significant degradation in both strength and elongation were observed after being subjected to extensive thermal ageing at 80 °C for 504 h. The proposed SBIG solder is promising for low-temperature applications.
